# The instrumental Brahmin and the “half-caste” computer: Astronomy and colonial rule in Madras, 1791–1835

**DOI:** 10.1177/00732753221090435

**Published:** 2022-04-23

**Authors:** S. Prashant Kumar

**Affiliations:** Humboldt-Universität zu Berlin, Germany

**Keywords:** Observatory, historical memory, colonial science, global history, scientific labor, human computers, time, caste, India, orphans

## Abstract

What did science make possible for colonial rule? How was science in turn marked by the knowledge and practices of those under colonial rule? Here I approach these questions via the social history of Madras Observatory. Constructed in 1791 by the East India Company, the observatory was to provide local time to mariners and served as a clearinghouse for the company’s survey and revenue administration. The astronomical work of Madras’ Brahmin assistants relied upon their knowledge of *jyotiśāstra* [Sanskrit astronomy/astrology], and can be seen as a specialized form of the kind of South Indian scribal labor and knowledge that also staffed the company’s tax offices. If at Greenwich the division of labor meant observatory work bore resemblances to the factory and the accounts office, in Madras, astronomy and accounting drew on similar labor forms because they were part of the same enterprise. But the company did not just adapt preexisting forms of labor, it also attempted to produce its own at a school built near the observatory to train “half-caste” orphans as apprentice surveyors and assistant computers. The school, staffed by the Brahmins, drew upon knowledge and pedagogical practice associated with the *tinnai*, the schools in which upper-caste children learned to read, write, and calculate. For a time, the observatory’s social order was literally “half-caste.” The paper also considers how the relationship between caste, status, and instrument was reflected in the visual and material culture of the observatory, such as in Indian-language inscriptions on its central pillar. For company astronomers, the measurement of time meant reworking the relationships among the Indian past, the colonial present, and an imperial posterity. Science under colonial rule spanned multiple temporal and social registers because it was the result of negotiations between the demands of political economy and the knowledge and practices of colonized others.

## Introduction

This paper is about the everyday practice of science under colonial rule at Madras Observatory. Colonial observatories make particularly visible the forms of hierarchy science was thought to require.^1^ They served as both centers of calculation and gateways to larger scientific networks for those at the margins of the imperial order, and so allow historians to ask fine-grained questions about how science scaled-up and scaled-out over the course of the nineteenth century. Applying for a post at Hong Kong Observatory in 1888, for example, an astronomer working at Markree Observatory in Ireland, William Doberck, cited his “experience in repairing instruments and also in employing uncivilized Irish peasants to help in scientific work.”^2^ By the end of the nineteenth century, the Government Astronomer at Madras complained that his “native assistants” were “machines without the certainty of machinery,”^3^ each “requiring perpetual control, more tedious than the actual personal labor.”^4^ Instruments and assistants became conjoined in the minds of observatory managers because both had to be regularly corrected to be reliable.^5^

As well as their labor, the knowledge and practices of “uncivilized” or “native” others were often assimilated into European science, sometimes unwittingly so. Darwin mentions, for example, in a discussion on sexual selection in *The Descent of Man*, that “the natives of Eastern Bengal” keep fighting birds known as bulbuls. He had heard from a contact in Calcutta that the males, being “pugnacious during the breeding-season,” tended naturally to “fight with great spirit,” which Darwin framed as an illustration of the law of battle in sexual selection. His contact, however, failed to relate that the bulbuls in question were especially trained to fight by the Nawab of Bengal and other Mughal elites, a tradition known as *shauq*.^6^ Knowledge from the colony did not pertain to a state of nature, though it was often assumed to. Darwin’s description naturalizes the bulbuls’ behavior because the imperial information order erased a “native” practice. A global history of science and empire would seem to require that we fragment the knowledge traditions of colonizer and colonized alike, and study the braided forms produced during moments of interaction.^7^

Historians have approached this problem by studying patterns of circulation within networks of global extent, following go-betweens, actors who, while often obscure, come to link larger circuits by crossing over from one physical or epistemic space to another.^8^ Studies following instruments and subaltern actors as the Great Trigonometrical Survey’s triangular grid expanded across India have established astronomy, for example, as an imperial field science.^9^ Such work has unsettled claims that “there is no evidence that physics or astronomy played any significant role in British imperial policy or colonial rule.”^10^ But in order to understand how the knowledge and practices of “native” others were absorbed into (and forgotten by) “Western science,” historians must pay closer attention to the colonial conditions of possibility for scientific labor.

Here I aim to provide a symmetric account of science under colonial rule by connecting scholarship on observatory practice in the history of science with studies of caste and representation in colonial South India. A growing literature has begun to examine how caste assumptions and practices shaped technological and scientific projects during British rule, highlighting the legacy of colonial education in converting caste privilege into social merit.^11^ I study the same topic at the end of the eighteenth century, before the “Orientalist” and “Anglicist” debates in the 1820s and 1830s surrounding systematic “native education” policy.^12^ In so doing, I hope to demonstrate that caste shows up in places like observatories because its forms of knowledge and hierarchy proved inherently compatible with certain kinds of scientific labor, such as observation and computation, as well as pedagogy.

In the first section of this paper, I show how changes in the political economy of Madras Presidency were reflected in the social order of the observatory, linking its astronomical work to surveying and taxation in the company’s Madras holdings. I argue that the role of “Brahmin assistant” emerged particularly out of South Indian forms of scribal knowledge and practice, also associated with the contemporary *kacceri*, or district tax office.^13^ Assistants in observatories were instrumental in a larger political-economic sense, but also in a more immediate social and interpersonal sense, as they performed rationalized forms of labor under strict instruction. Ravi Ahuja has argued that the company sought out “*ancien régime* forms of subordinating labor that proved to be compatible with colonial conditions.”^14^ Nevertheless, how these labor forms were modified as they were integrated into colonial scientific institutions remains poorly understood.

In the second section, I examine records of a school at the observatory built to train “half-caste” orphans as apprentice surveyors, where the Brahmins taught using methods drawn from Tamil schools.^15^ Conflict between the observatory’s Brahmin assistants and the boys of the Survey School shows how structures of race and caste were made to mesh in organizing scientific labor in the colonies. I focus on a single site in order to show that, when the issue of colonial compatibility was worked out in practice, new ways of ranking and dividing labor emerged as interpolations between race and caste. The third and fourth sections relate Madras’ changing political economy to the novel linkages between Indians and instruments that emerged in the colonial archive in the first decades of the nineteenth century. The relationships among caste, status, and instrument were reflected in the textual archive, as well as the visual and material culture of the observatory. Traces and margins, like the Indian-language inscriptions on the observatory’s central pillar, show that the observatory was also made to signify in a local register. I argue that science in the colonies should be understood as heteroglossic, multiauthored, and intended to signify across several social and temporal registers simultaneously. Measuring time was both a technical and a historical project, concerned with reworking the relationship between past, present, and posterity, but it was only possible through often asymmetric negotiations with local forms of knowledge and practice.

## A collectorate for the stars

Madras Observatory was built in Nungambakkam, in 1791, at a spot near the Cooum River “defended by the Guns at Egmore Fort.”^16^ Michael Topping, a marine surveyor, was made the first Company Astronomer.^17^ The observatory was to be “a point in India from whence a ship may calculate the longitude with as much safety as from the best known land mark on the coast of England.”^18^ In this kind of astronomy, observations were used to fix the coordinates of a large number of stars on the celestial sphere. Once mathematical corrections were applied to this data, to account for orbital and atmospheric effects, the predicted timings of astronomical phenomena made it possible for mariners and surveyors to safely reckon the lunar and planetary motions necessary for determining longitude and latitude. The Company provided funds for the observatory building, and an assistant, John Goldingham (formerly known by his Danish, and probably Jewish, surname, Guldenheim), who was also appointed master of a surveying school, housed near the observatory^19^. Like Greenwich, the observatory was to provide local time and ephemerides to mariners, and by 1802 it marked a geodetic baseline for surveyors.

A year after its founding, a “Bramin” known as Srinivasacharya Braminy was hired to assist in the observatory and to teach in the school.^20^ By 1808, there were two Brahmin assistants, whose “respective duties were nearly alike; only one [Srinivasacharya], having more experience than the other [Verdacharya], observed more, and had greater advantages from speaking and writing better the English language.”^21^ The Brahmin assistants were tasked with the observations and computations required to regulate the observatory’s central clock.^22^ Their capacity for rapid, complex calculation could be readily fit into a technical order in which the sophistication of mathematical technique was increasingly seen as “admirable artifice[s] that, by shortening computations, extends astronomers’ lives [just as] the telescope ha[s] increased their sight.”^23^

Caste-based patterns of recruitment undergirded the early company knowledge economy as a whole, most notably in the case of language education, which provided access to subcontinental epistemic spheres like astronomy [*jyotiśāstra*] and jurisprudence [dharmaśāstra].^24^ The Calcutta jurist William Jones, for example, relied upon his Sanskrit-Persian *munshis* [language teachers] for their linguistic knowledge and familiarity with socioreligious texts, as well as his own knowledge of biblical criticism, in formulating the notion of an Indo-European language family.^25^ In the South, Thomas Trautmann has identified, in the first decades of the nineteenth century, a “Madras School of Orientalism,” associated with Colin Mackenzie and the Dravidian philology of the College of Fort St. George. He has shown how the proof that South Indian languages like Tamil and Telugu did not derive from Sanskrit relied upon both vyākarana, or Sanskrit grammatical analysis, and biblical hermeneutics, the latter contributed by Francis Whyte Ellis, Collector of Madras, and the former by Sankaraiah, Ellis’ Telugu Brahmin *sheristadar*, or chief accountant.^26^ The relationship between Madras and the center of British power in Calcutta was unequal, with “no thought . . . that Madras would displace Calcutta, whose supremacy was secure, only that Madras would amend Calcutta’s errors.”^27^ Madras Observatory offers a contrasting case study to language education, since it was founded at a time when there was no corresponding observatory in Calcutta, and matters of practice could be decided according to South Indian circumstances, independently of any Bengal precedent.

Brahmins hired into observatories also differed from language teachers in that they were employed by a permanent establishment, of which they were frequently given sole charge. Company astronomers, usually out on surveys across South India, left most of the actual observatory work to the assistants. It was their labor that kept up the chronometric regime of Madras harbor. The heat in India caused brass to expand, changing marine chronometers’ rates and making standard methods of longitudinal navigation difficult or impossible without repair.^28^ The rewinding and return of ships’ marine chronometers was tasked to the observatory’s Brahmin assistants. Fixing chronometers was also a scribal practice, with measurements of rates and meteorological observations recorded and archived by the Brahmins.^29^ From the 1790s, East India Company ships’ logs carried a column for chronometer ratings, a reflection of such structures of maintenance.^30^ Demand for numerate labor to observe, compute, and correct clocks was caste-bound just like language education. Observation and computation required skills in literacy and numeracy also possessed by other groups employed by the company, such as *kanakkupillai*s, or scribes in the revenue administration.^31^

In the Company’s district revenue offices, known as *kacceri*s, the state of constant data production demanded by bureaucratic oversight was maintained by South Indian scribal skills and knowledge.^32^ A *kacceri*’s *sheristadar*, the arch-collaborator who oversaw Company accounts in so many collectorates across the presidency, was almost invariably a Telugu Niyogi or Marathi Deshashta Brahmin.^33^ They were typically familiar with the languages of record: Persian, Telugu, and Tamil. Underneath them worked an army of *kanakkupillai*s, stratified by caste and language, with Tamil Brahmins comprising the uppermost layer.^34^ The relative power of the *dubash*, which meant a man of two languages, a relatively heterogenous category of power broker throughout much of the eighteenth century, had begun to diminish toward the end of the eighteenth century, as the Company began to recruit upper-caste Hindus directly into its administration.^35^ Slowly, free agents, go-betweens, and contractors were subsumed by an expanding administration, in which caste hierarchies came to determine labor hierarchies. Company officers began to rely heavily on *kanakkupillais* in constructing the “document Raj” required to carry out revenue assessment and extraction.^36^

The observatory sat at the intersection between the emerging “Madras School” knowledge economy and the expanding revenue administration in South India: until 1810, the offices of the Inspector of Revenue Surveys and Company Astronomer were held conjointly. In India, positional astronomy was used to coordinate revenue surveys.^37^ In the Kolar district, for example, the first such survey, conducted in 1794 by Alexander Read, resulted in a twenty-one percent increase in land revenue.^38^ The observatory was intended to serve as an inspectorate and clearinghouse for the work of surveyors in the field, with copies of maps and data archived in the observatory building itself.^39^ If this new regime was met with recalcitrance from surveyors, it encountered outright resistance from hereditary landowners. In 1794, for example, a group of Brahmin landlords in Baramahal refused to witness the measurement of their *agraharams*, or hereditary lands, by Company surveyors. Beginning in 1816, Governor of Madras Thomas Munro’s *ryotwari* system of tax collection attempted to refine the system, and to use the surveying establishment to directly measure portions of land for which cultivators, the *ryot*s, would be taxed.^40^

From its founding, then, Madras Observatory can be understood as a kind of collectorate for the stars, its assistants in effect stellar clerks.^41^ The observatory’s “native assistants” were drawn from the same Brahmin communities as staffed the revenue administration. Its social order thus in many ways resembled an accounts office.^42^ Astronomy and mathematics were known by the British to be caste-bound forms of knowledge, with only Brahmins permitted to learn and practice *jyotiśāstra*, the branch of Sanskrit knowledge dealing with astronomy and mathematics.^43^ We know from a Company Astronomer’s (failed) attempt in 1808 to have Srinivasacharya and second assistant Verdacharya translate the *Surya Siddhanta*, a canonical Sanskrit astral text, that the first two assistants had expertise in *jyotiśāstra*.^44^ Experts in *jyotiśāstra* and scribes alike were part of the urban service gentry, which saw significant expansion during the first decades of the nineteenth century.^45^ In Company service, caste became tied to rank, a pattern replicated by subsequent meridional observatories at Colaba in Bombay and Chowringee in Calcutta.^46^

## Blood and education

In the Tamil-speaking regions of Madras Presidency in the eighteenth century, Brahmins, as well as the scribal and cultivating castes, were typically educated at *tinnai* [verandah] schools in each village, named for the raised platform outside a house where lessons were typically held. Children became students usually around age five, but progression through the curriculum – which included the Tamil alphabet, arithmetic, fractions, and rudimentary geometry – was based on students’ aptitude for memorization and recitative exercises. The system was a monitorial one: more advanced students were instructed by a tutor, and in turn taught those less advanced. Students would recite tables and verses from memory while simultaneously writing them out on a palm leaf, using an iron stylus. Multiplication tables were committed to memory through recitation, with the sequence of arithmetic operations required for various tasks (like conversion between grain measures, or the measurement of land area, both calculations essential to Company administration) remembered with the aid of a mnemonic verse. Nevertheless, those who actually carried out the physical measurement of land in the traditional Tamil revenue bureaucracy, which the *ryotwari* system partially absorbed, were not admitted to the *tinnai*, because they were of lower caste.^47^ For further education, elite groups like Tamil Brahmins and Telugu Niyogi Brahmins flocked to small schools that offered instruction in the English language.^48^ By attending the *tinnai* and then learning English, upper-caste children could hope to acquire knowledge and skills that would be useful in the Company administration.

Topping had also founded a surveying school, housed near the observatory, to meet the need for revenue surveyors in the field. Before its closure in 1810, the school trained forty-two boys, most of whom were the mixed-race orphan sons of Europeans, usually aged eleven or twelve when first apprenticed.^49^ Goldingham was appointed master, but most of the classes were taught by the observatory Brahmins. Srinivasacharya, like Verdacharya, is a name affixed with a title, *ācārya*, marking one a teacher, one who has undertaken Sanskrit learning. They taught the Tamil and Hindustani languages, methods of land and water surveying associated with the *tinnai* system, and European methods of surveying when Europeans were absent. The students were drawn from the Madras Male Asylum at Egmore, established by the Scottish pedagogue and clergyman Reverend Andrew Bell, who taught using a modified version of the *tinnai* model. Bell rechristened it the “Madras system,” crediting the *tinnai* only with the idea of teaching students the alphabet and basic arithmetic using figures drawn in sand.^50^ Ironically, the *tinnai* schools of Madras supplied the nucleus of pedagogical practice around which the Survey School grew.

Madras was the first city in the world to make the color line literal, with a stone wall dividing the European and (primarily lower caste and working class) Indian districts, respectively named “White Town” and “Black Town.”^51^ That the children of the Survey School could move across this line made them both utile and suspect. But Topping held that the properly instructed offspring of Europeans could be made useful to the Company, “instead of being suffered to fall a sacrifice to Idleness and a vicious course, as most of them, it is to be feared, will do.”^52^ This apparently natural tendency was believed to be the poisonous effect of native mothers: Reverend Bell said he saw “in the very first maxims which the mothers of these children instil into their infant minds, the source of every corrupt practice, and an infallible mode of forming a degenerate race.” The result was that “the half-cast children of this country shew an evident inferiority in the talents of the head, the qualities of the mind, and the virtues of the heart.” Bell’s “Madras system” promised to make even such suspect hybrids into useful company instruments.^53^

Still, most in the revenue administration preferred European surveyors. Topping argued this made no economic sense. Every European would require an interpreter, lest he be cut off from all local news; ample free time; and a long period of seasoning, during which illness and death were common. Moreover, he complained, importing a single European would cost six times what it would take to train a country-born observer. Instead, the Company could use labor latent in orphanages to train their own intermediaries. Topping told the Madras government at Fort St. George that “the advantages of having a body of men, were they to be employed merely as interpreters, who being allied to us by blood and education would form a check upon the Malabar natives [Tamils], whose language and customs are as familiar to them as our own, is too obvious to need many words to be spent in favor of it.”^54^

The most promising of the Survey School boys also assisted in the observatory. Their presence meant matters of trust, skill, and race were at issue in the allocation of computational labor. The Brahmins were limited to corrections due to errors in clock time, which did not require the logarithmic methods British astronomers used in applying corrections for refraction and orbital effects like precession. The “long and skilful computations” required to produce publishable observations were “always carried out by myself and two of the most advanced boys in the school.”^55^ Error correction could only be performed by, as Topping had put it, those “allied to us by blood and education.” Prestige conferred by descent made the work of the “half-caste” computers trustworthy, and it was the invidious performance of such skill that served as a “check upon” the instrumental Brahmins.

The resulting social order was contradictory and unstable, with the Brahmins holding rank over the boys in the school but subordinate in the labor order. The situation eventually came to a head. At the end of 1810, the Survey School was abolished, a casualty of the reform of the survey administration along hierarchical lines, which eventually elevated Colin Mackenzie to Surveyor General of India in 1816.^56^ The secondary positions the Company Astronomer had held, as Inspector of Revenue Surveys and Survey School Superintendent, were both abolished upon Mackenzie’s promotion. In the resulting power vacuum, relations between the Brahmin assistants and the boys of the Survey School became particularly fraught, and therefore visible. The earliest surviving record produced by Srinivasacharya is a letter of complaint, sent to the Company Astronomer, John Warren:

It is with much regret that, induced by constant trouble and vexation from Mr Scott and Mr Bailie, I state my humble case before your honor, which has been one of continued trouble, ever since the school was taken from under your charge. If I have been so long without doing it in writing, it was in obedience to your orders, as you above [all] forbid me to bring you complaints, and directed me to go to Lieut Col. Mackenzie.

For the last four months there has been scarcely one day but that I and fellow Bramin assistant have been kept in fear by Mr Scott’s threats to get us turned out. Almost every day, boys from the school, whilst it stood, came and steal stationery and pencils and which I complain to Mr Scott he laughs at me and never gives no redress.

Like many pupils of the Survey School, nothing is known about William Scott’s parents. He attended the school from 1798 until 1801, when, aged fifteen, he went with Warren on the first survey of Mysore, carried out in the wake of Tipu Sultan’s defeat two years prior. Previously employed as an assistant master in the Survey School, Scott had lost the rank upon the school’s abolition. And according to Srinivasacharya, he’d started to drink:

When Mr Scott gets in liquor he is some time very violent and abusive to me and fellow assistant; sometimes threatens to beat me though he has never done so out of fear of your honor. He says that Verdachary is no Bramin of observatory though he has seen him teaching the boys for more than six years the Hindostanee in the School and attend regularly every day in the observatory winding the watches registering transits and rate.

Mr Scott came one day to the observatory just after the school was abolished and told me in presence of Mr Bailie, Bramin this is the time for you to cheat with impunity to sell some sextant out of the observatory, because as it is to be abolished in the confusion it will not be perceived.^57^ I answered him that he had better mind what he said, that a Native could have as much honor as an European, and that he would not dare to say so much to a man of his own cast.

On the 3rd of the month after you had found fault with me for having removed the rain gage and I told you I had done so by Mr Scott’s desire – Mr Bailie called me in the evening into his room where was also Mr Scott. Mr Bailie seemed intoxicated. He came to me speaking very loud but so confusedly, I could not hear a word distinctly, I only understood that he said that I had told a lie to Captain Warren about Mr Scott, that I was a rascal for having so done, and he rose two or three time from his chair as if to flog me, putting his fist under my nose, and Mr Scott was laughing all the time encouraging Mr Bailie who was in liquor and who came to complain to your honor immediately.

Sir, it is impossible we can go on in peace being so constantly disturbed by these young Surveyors who leave [sic] close to the Observatory, for every time they get in liquor, which now happens very often since they are no longer under your honor’s Protection, we are sure to be troubled, sometimes by threats to get us turned out, and often by abuse of your honor’s person which is more to their shame, as before they always say your honor was a father to them.^58^

This episode is important because it gives us a glimpse of the observatory’s social order in the absence of European superintendence. The proper functioning of the observatory relied upon its caste order being subordinate to, but otherwise relatively independent of, its racial order. But the inherent vice in Topping’s plan for the Survey School was, in a moment of crisis, exactly its touted advantage: that “half-castes” were both Indian and European. When the Survey School is closed, the boys, no longer bound paternally and financially to the Company, freely and drunkenly confound caste and rank. While Srinivasacharya was paid 35 pagodas a month (just under a fifth of the 192 pagodas Warren himself earned), Verdachary, as second assistant, was paid some 25 pagodas, equal to the salary of assistant surveyors like Bailie and Scott.^59^ Scott denies Verdachary is an observatory Brahmin because he and the Survey School boys shared the same rank, if not the same caste. Significantly, the Survey School boys are among the first to be described as “half-caste” in print, their positionality first theorized at a site of scientific labor. The children so described were educated in European surveying methods by Tamil Brahmins, who themselves taught in an appropriated version of the *tinnai*; it was really the system itself that was “half-caste.” Therefore, the early Company knowledge economy not only selectively preserved existing forms of subordinating labor, but in fact produced its own, at the interface of caste and race.

## Trust, hierarchy, and the colonial archive

After the closure of the Survey School, the observatory was subordinated to Colin Mackenzie’s survey, and assistance was provided solely by upper-caste Indians. Brahmins became all-purpose computers under a later, much more intensive labor order.^60^ T. G. Taylor, who succeeded Goldingham in 1830, resolved to begin an expansive program of astronomical observation, comprising observations of some 11,015 stars, taken from 1830 to 1843, which would later result in the Madras General Catalogue. In order to process the huge number of astronomical and meteorological observations required, the observatory relied upon, and made invisible, the arithmetic capacities of the observatory Brahmins. However, calculating the various reductions that turned “rough observations” into a publishable catalogue was a different sort of activity than either rewinding watches or noting the time of transit. Because reducing an observation took four or five times as long as taking the observation in the first place, leaving some ten thousand observations unreduced was to render them essentially useless. Taylor was well aware of this, and opens his catalogue by quoting the Astronomer Royal, George Biddell Airy, at length, on the moral duty an astronomer had to reduce his own meridional observations.^61^

Trust in the Brahmins was therefore never total, at least not in print. Proper error correction “was an undertaking attended with considerable labor and anxiety, especially as the Native Assistants being very liable to blunder, I could expect from them but little assistance.” In 1831, Taylor “found it necessary to examine carefully every computation made by the Native Assistants, or to procure from them a duplicate computation.”^62^ A year later, by 1832, Taylor was more confident in his assistants. “As computers,” Taylor reported, “[native assistants] possess a very serviceable degree of accuracy.” Still, he said he “trusted nothing of importance to the native computers without a strict examination or recomputation.”^63^

Taylor’s imperious yet short-lived frustration with his Tamil computers suggests they quickly developed modes of translation that made it possible for them to “show their work” in reducing observations. Methods of representing and working with numbers common to British observatories were not incommensurable with *tinnai* methods; besides logarithms, the core mathematical corpus was in common. The routine work of reducing observations would have required assistants familiar with the most advanced *tinnai* texts, which dealt with methods in *jyotiśāstra*, on sexagesimal fractions, spherical trigonometry, and infinite series. By far the largest portion of the work would have been elementary arithmetic, every printed observation requiring dozens of individual computations, carried out mentally. But technical complications must have been considerable; for example, the contemporary Tamil number system, as described in the *Ponnilakkam*, was divided into three “layers,” consisting of the ordinal numbers, fractions in units of 1/320, and fractions consisting of a 1/320 part of 1/320.^64^ Even basic calculations would have required the computers to function as mathematical *dubashes*[translators], translating between Tamil and European arithmetic convention. Taylor’s catalogue is therefore a kind of braided knowledge, but here the knowledge of the colonized is made to do invisible labor only, and never to determine the questions.^65^ For his efforts, Taylor was lauded by the Astronomer Royal as having produced “the greatest catalogue of modern times.”^66^ Taylor’s total control over all aspects of the catalogue’s production, made possible by his Brahmin assistants’ capacity for mathematical translation, was the guarantee of its quality and value to the science.

Between Madras Observatory’s founding in 1791 and its closure in 1899, every head assistant was Brahmin. The proper working of the observatory relied upon the reproduction of Brahminical norms and proscriptions within a larger, British bureaucratic-imperial order. When Srinivasacharya Braminy retired in 1830, for example, he was granted both a pension and a palanquin, the latter an honor typically afforded to the head of a Hindu temple. The Company proceedings announcing his retirement identify him only as the “Head Assistant,” his caste implicit in the rank and so never mentioned.^67^

While service gentry like Srinivasacharya receive direct (if misspelled) mention, it’s an enduring irony of the colonial archive that we often hear about the marginalized in the literal margins of official documents. One may trace this by looking to the footnotes and remarks studded throughout Taylor’s catalogue, indicating observations likely to be in error, due most often to weather, or, on one occasion, “a fine thread which had been attached to the pendulum [of the observatory clock] by some mischievous spider.”^68^ Only in these notes is there any mention of those among the lower stratum of observatory hands, mostly lower caste “peons” or “lascars,” and employed on a casual basis by the Brahmin assistants. Usually a transparent part of the observatory, the high-caste assistants’ own assistants only appear in the record – like Boyle’s invisible technicians – when something goes wrong.^69^ If the Brahmins received greater scrutiny under Taylor, so too did others lower in the hierarchy.

One morning the head assistant found the transit wires broken and identified the culprit as an assistant named Samian, who had been allowed to sleep in the observatory by the *chowkidar* [gatekeeper]. “Curiosity,” the head assistant wrote, “I apprehend had prompted him to finger and consequently to break the wires.”^70^ The English spelling of the assistant’s name is unusual. Tamil is a highly diglossic language, its spoken register differing markedly in orthography and several other respects from its literary register. The latter was taught almost exclusively to upper-castes. The head assistant uses a Romanization of the Tamil literary form of the typically lower-caste name Samy [cāmiyin]. “Samian” is probably the result of the high-caste assistant transliterating his own notes into English for the European superintendent. That a subaltern assistant should be referred to by a name he would almost certainly not have used to refer to himself bespeaks the essentially Brahminical nature of the social order at the observatory.

The transit wires were on another occasion subject to deliberate interference. On March 23rd, 1831, for example, we learn, from the head assistant’s notes in the Transit book, quoted in Taylor’s catalogue, that “a piece of wood about a quarter inch long appeared suspended on the 4th wire [of the transit instrument] which with others it had broken.” Before commencing observations, the Brahmin assistants would first string spiderwebs across the eyepiece of the telescope, spaced at regular intervals, to serve as a reticule. To break the threads was to ruin an entire day’s observing. A week later, a similar interruption occurred. The second happening invited suspicion, and greater scrutiny: “This piece of wood . . . had evidently been recently picked, the ends shewing the fibres to have been twisted in the act of being broken from the tree by the miscreant hand who introduced it, and the bark on being removed exhibited the sap in a moist state; I am quite at a loss to account for the motives of the individual who can thus act!” In a footnote written by Taylor we learn that he had “great reason to believe that an Invalid Sepoy to whom the keys of the Observatory were intrusted [sic] during the absence of the Assistants, is the person who introduced the pieces of stick abovementioned into the Instrument; he was (as I afterwards found out) occasionally intoxicated and was in consequence discharged.”^71^ To place a twig on the transit wires wasn’t an outright act of sabotage, but it did stop the daily observations, which the Brahmins ultimately had to answer for. The sepoy was moreover likely a man of lower caste.^72^ If indeed the sepoy did tamper with the transit instrument’s reticule, his motives are not as opaque as the author of the footnote suggests.

**Figure 1.**
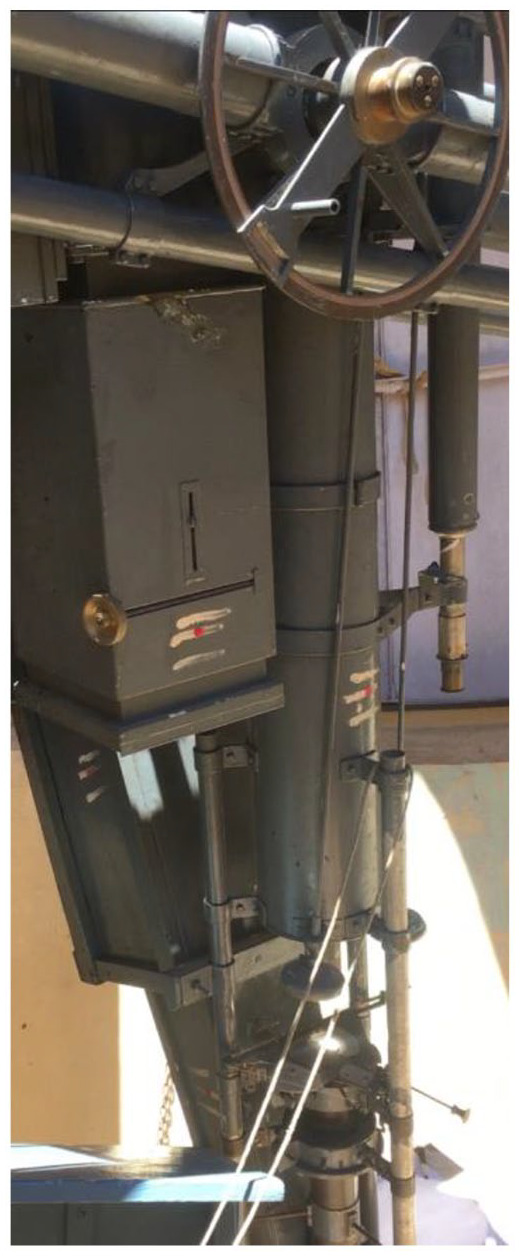
The transit telescope at Kodaikanal Observatory. Author’s own work, February 2020.

The caste-bound restrictions over space in the observatory parallel those of the Hindu temple. The transit instrument sat at the observatory’s *garbagriha*, or *sanctum sanctorum*: the temple’s central room, which housed the idol. The *garbagriha* was barred to all but priests; similarly, the sepoy wouldn’t usually have been admitted to the central room housing the transit circle. Repeatedly messing with the transit wires suggests the sepoy was familiar with the usual preparations for observation. The offending twig is a contaminant that stops the ritual; it functions like a chicken bone in the temple *prasadam* [vegetarian offering]. The sepoy’s act, in contrast to “Samian’s,” was interpreted by the high-caste assistants as both an act of trespass and the desecration of an idol. The caste-ordering of scientific workspaces appears in different guises down to the present day.^73^ Indeed, the telescope attached to the transit circle at Kodaikanal Observatory (Figure 1), Madras’ successor institution, is painted with a *tilak*.^74^

## Representing labor and time

The instrumental Brahmins I am concerned with in this paper were never a dominant trope; Europeans usually reported that the Brahmins’ predilection was for the abstract. The French savant and traveler Le Gentil in 1780 reported that Nana Motu, a Tamil Brahmin at Pondicherry, could calculate the time of an eclipse in about forty-five minutes, far more quickly than was possible using European methods.^75^ This was all the more remarkable since jyotiśāstra made no use of the telescope. Where Brahmins used instruments, they were limited to the *gnomon*, a rod used for recording shadows, the astrolabe,^76^ and the masonic mural instruments built by Jai Singh II in North India.^77^

Nor were they usually regarded as wanting anything to do with European instrumentation. A widely serialized story about a Brahmin and a microscope, first published in 1813 but drawn from the artist James Forbes’ memoirs of the period 1765–84, styles the sacerdotal caste as “fifty millions of people . . . all happy in their ignorance!”^78^ The story follows “an English gentleman, extremely fond of experimental philosophy,” who befriends a Brahmin “who read English books, searched into the encyclopaedia, and profited by the best philosophical instruments.” One day the Englishman receives a microscope as a present from Europe. He immediately “showed it with rapture to his Hindu friend and in opposition to the scheme of the metempsychosis, discovered to him the innumerable animalculae devoured by the Brahmins on every fruit and vegetable they eat.” The Brahmin grew despondent, and retired without explanation. When next they saw one another, the Brahmin made repeated overtures to purchase the microscope. The Englishman eventually relented, whereupon the Brahmin seized the instrument, “descended with a quicker motion than is customary with his caste,” and smashed it upon a rock in the garden. He begged the Englishman “convey no more implements of knowledge and destruction!”^79^ Given the wide currency of such stereotypes, it is somewhat surprising to find Brahmins hired to mind instruments.

**Figure 2.**
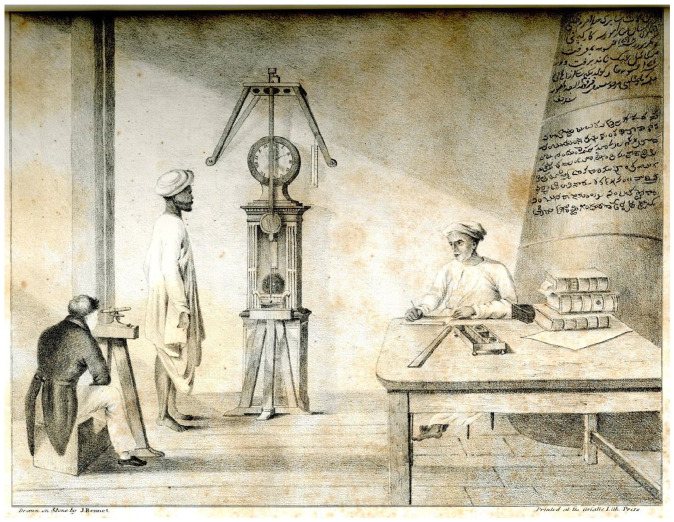
Goldingham performing a pendulum experiment with Srinivasacharya and Tiruvenkatacharya. From John Goldingham, Madras Observatory Papers, Calcutta: Asian Lithographic Press, 1826. www.dspace.cam.ac.uk/handle/1810/238682.

Instrumental Brahmins are depicted in the colonial archive as part of a project of stabilizing a new representational economy, in which certain kinds of Indians were depicted as trustworthy so that the knowledge claims of their European superintendents could be taken seriously. They appear in a lithograph sandwiched between tables of metrological observations. Shown in Figure 2, the image depicts three figures, arranged around a complex experimental setup, sometime in March of 1821. Goldingham, now Company Astronomer, is the tailcoated European inserted in the shallow foreground. He works with two Brahmin assistants. Tiruvenkatacharya, standing, is the younger of the two, his manual disposition evidenced by his hiked-up *dhoti*. The seated assistant is Srinivasacharya. A Vaishnavite *tilak* is just visible on his brow, and marks him as an Iyengar Brahmin. The pair are the first Indians to be credited in the Royal Society’s imprint, the *Philosophical Transactions*.^80^ The three are conducting a gravimetric pendulum experiment. The pendulum, whose period could be adjusted by changing its length, is mounted to a frame, so that it hangs in front of the grandfather clock below, its knife-edge pivot point sitting balanced on agates. It was sent from London to Madras by Captain Henry Kater, a surveyor returned from India himself, and now “one of the most active Commissioners for the enquiry into the state of the Weights and Measures appointed by H.M.’s Government.”^81^

The pendulum shows up in Madras because it provides an experimental means of relating fundamental quantities, of registering space in terms of time. It was known that the period of oscillation of a pendulum is proportional to the square root of a constant that depended on the pendulum’s length and the local acceleration due to gravity. The task is to find the length of the pendulum that will cause it to oscillate once in two seconds. In order to measure its oscillation period, the hanging pendulum is suspended so that it sits directly in front of the grandfather clock’s pendulum, the latter’s rate having been determined by comparison with the observatory’s astronomical regulator. Of Kater’s own invention, the length of the seconds pendulum was to be the standard for fixing the yard as a unit of measure.^82^ But since the length of a pendulum beating seconds was known to vary across the earth (due to the planet’s variable density and curvature), a metrical standard required measuring the length of the seconds pendulum at the farthest ends of British dominion.

As Kapil Raj has noted, during field surveys and other exercises in the mapping and domination of space, the bodies of Indian go-betweens were turned into measuring instruments.^83^ The observatory also relied on the strict instrumentalization of its Brahmin assistants’ capacities. The experiment relies on the Brahmins registering numbers when called upon by Goldingham. By counting the number of coincidences between Kater’s pendulum and that of the grandfather clock, the period of Kater’s pendulum could be very accurately determined.^84^ So as to avoid parallax error, the pendulums are under the gaze of Goldingham’s theodolite. He is watching for the coincidences. When he notes that the pendulums are in sync, he calls out to Tiruvenkatacharya, who then counts the seconds until Goldingham next sees the two pendulums swing in synchrony. Since each trial took up to an hour, the experiment depended upon coordination between Goldingham and Tiruvenkatacharya; he tells the Royal Society that “the youngest Bramin assistant” registered the clock “with great correctness.” Srinivasacharya, the first assistant hired by the observatory, was by now advanced in age, and had been charged with recording the numbers read out to him by Tiruvencatacharya.

Goldingham could reconstitute Kater’s experiment because the Brahmins were largely familiar with contemporary European experimental practices from their usual observatory work. The image is thus an argument: if this experiment could be replicated all across the planet (and all perturbing errors corrected for), one could, in theory, assemble a global picture of gravimetric variation.^85^ Moreover, it captures and communicates what made science in the colonies possible. Under colonial rule, hierarchies of labor and lines of erasure depended on race, caste, and class. Thus we know that in Madras, two European workmen, a Mr. Bruce of the dispensary and a Mr. Gordon, jeweller, were consulted in measuring the effect of the buoyancy of air on the pendulum. The Brahmin assistants are identified, but only in a footnote, and we are left to infer that the details of the experimental *mise en scène* relied on anonymous Indian technical skill. Although Goldingham disparaged “the little solid assistance to be obtained from workmen in this country,” it was on local technicians that the niceties of experimental fabrication relied – the grinding of agates till spherical, to make a pivot point for the pendulum, for example.^86^

Kater had carried out his experiment in 1818, in the basement of Henry Browne, a Fellow of the Royal Society and “late Chief of the East India Company’s Settlement at Canton in China.” Browne’s London house was “situated in a part of Portland Place” (a wealthy street, built unusually wide) “not liable to much disturbance from the passing of carriages.”^87^ The success and subsequent publication of the results of the pendulum experiment suggest, therefore, that artisans in 1820s Madras could support experimental endeavors as sophisticated as those in contemporary London. For a time, the farthest flung metrological regime in the British Empire straddled two unlikely venues: a basement in London and an observatory in Madras. Goldingham’s intent was to use Madras as a metrological platform, carrying out the same experiment on an equatorial island off the coast of Sumatra.^88^ In the early part of the nineteenth century, the universality of fundamental quantities was slated to depend on the East India Company’s control over time.

Looming over the three men in Figure 2 is a pillar, inscribed with Persian and Telugu text. Where the experiment was intended to establish control over space, the pillar signified control over time: past, present, and future. The pillar supported the observatory’s transit instrument, and was the means by which the Brahmins produced local, sidereal time for the city of Madras. Topping placed a granite tablet over the western door of the observatory, similar to one laid at Greenwich, bearing an inscription marking the site “devoted to astronomy at the cost of the English Company trading to India, under the patronage of Charles Oakley, commander of Fort Saint George.”^89^ “An inscription to the same purport” was to be “engraved on the Cone in the center of the building in the three principal languages of the country,” that is, Persian, Telugu, and Tamil. The inscriptions were to be the means

by which (as the lapse of many centuries makes but little impression upon this Granite) Posterity may be informed—a thousand years hence—of the period when the mathematical sciences were first planted, by British liberality in Asia. May they take deep root, and branching forth into every useful, every elegant art, long prove a blessing to the future generations of this extensive Empire; and an honor to the great European Power that now governs it, with such distinguished Glory and Felicity!^90^

The Telugu text, although archaic and difficult to make out in full, does appear to be an approximate translation of Topping’s words above. It is no accident that the languages inscribed on the observatory’s central pillar are those used for official purposes by the *kacceri*. The inscriptions were added to the lithograph by a practiced, and very likely Indian, scribal hand. The text should be understood dialogically, as Topping’s translation of the plaque into an upper-caste South Indian register, styled after the inscribed granite stelae that marked Vijayanagar-era (c.1336–1650) land grants. Irschick borrows the term “dialogic” from Bakhtin to analyze the production of history and identity in colonial South India by Indian and European voices, which are not always distinct or cleanly separable. For Bakhtin, speech is dialogic, or heteroglossic, when it represents “another’s speech in another’s language, serving to express authorial intentions but in a refracted way.”^91^ In inscribing Topping’s words on the pillar, natives are made to accede to the colonizer’s claim to have begun mathematical history on the subcontinent.

Topping was aware of the importance of the epigraphic register to historical memory in South India, justifying the pillar’s expense to Company directors by noting that granite was “used on all works of Magnificence in this Country.”^92^ He was writing history in two registers. As he told the East India Company Directors in London,

astronomy, it is true, had been cultivated by the ancient Inhabitants of these populous regions, from times immemorial; but because little now remains of what was achieved in those very remote Ages, except a few imperfect Tables . . . of slight import to the furtherance of the Science itself, which, before the invention of the Telescope and the Pendulum-Clock, could never have arrived at any very high degree of improvement . . . it became necessary to recommence the work, with all the advantages [afforded by] the present improved and improving state of sciences in Europe.^93^

The pillar supported an advanced telescope but was constructed and inscribed according to South Indian convention. The Company established the authority of their science in India by claiming to be “recommencing the work,” speaking in a self-consciously ancient, appropriated, register. The pillar signifies that the observatory was endowed and maintained by the latest dynasty able to intervene in the epigraphic record, the East India Company. The very fact of the observatory’s founding was reckoned a claim over astronomy in Asia; the Company had not only invaded and conquered a territory, but also an epistemological space.^94^ The central pillar at Madras Observatory is a material relic of the rescaling of historical time laid down in Topping’s plan: it serves to rebraid the relationship between the past “Ages” to which Brahmins’ astronomy belonged; the sidereal present, which they are tasked with maintaining; and an imperial “Posterity . . . a thousand years hence.”

And yet without the continuity of the mathematical sciences on the subcontinent, the observatory’s particular labor regime would have been impossible. It was intended that, when all had fallen to ruin, the self-image of empire, as having come bearing mathematics, would remain.^95^ “Native” skill and knowledge are written out of the history of science in the same moment they are made useful for empire. The Brahmins, just like the pillar, bear and maintain modern instruments, but are wholly identified with an ancient past. Company astronomers styled themselves as continuing the astral sciences in the subcontinent, thereby conferring ancient prestige on the modern observatory sciences, while simultaneously yoking the Brahmins to the instruments they were hired to operate.

While relatively poor Brahmins may have been instruments in the observatory, those possessed of land and capital were instrumental in the consolidation of Company power. One sense of the word “instrument” slips easily into another in the colonial archive. The few wealthy Brahmins who circulated through the Madras Observatory were afforded certain privileges not offered to those among the rank and file assistants. The case of Goday Venkata Juggarow is instructive. The scion of an Anglophile family of wealthy Telugu Niyogi Brahmins, Juggarow’s father and uncle were *zamindars*, hereditary landowners who collected taxes on behalf of the company in Vizagapatam, north of Madras. In 1834, aged seventeen, he began to study astronomy under Taylor in the observatory and was afforded privileges beyond those of the Brahmin assistants. Eighteenth months after commencing studies, he published a table that when used “with the assistance of the Nautical Almanac will afford a ready means of discovering when occultations will happen.” This was a performance for a largely, though not exclusively, British readership. Juggarow’s table meant that “persons possessed of a Telescope and Regulator will be enabled to make the longitude to a very great degree of accuracy.”^96^ Few Indians outside the elite circles Juggarow himself traveled in could claim to belong to such a group. In 1836, a second paper by Juggarow appeared, on the mass of Jupiter.^97^

Though admittedly precocious, Juggarow’s contributions were included in the *Madras Journal of Literature and Science*, “not because we assume it will inform the Scientific Astronomer of anything he was unacquainted with before,” but rather “as a *literary curiosity*, being the production of a native of India, among whose brethren intellectual efforts of such character are too uncommon, to pass by the present one.” By a transposition of genre, the “native” is written out of the history of science: Juggarow’s astronomical contributions become part of a literary history of conquest, produced by British tutelage. He was the first of several Indian astronomer-princes the British would patronize in the nineteenth century.^98^ The journal’s editor, Robert Cole, noted that Juggarow “promises to be a distinguished instrument in the good work of elevating the people of India from the character of indifference to intellectual acquirements, too justly chargeable on them, we are sorry to say, and those of the Peninsula [South India] especially.” Thus the observatory could also function as a site for the production of civilizing instruments, elite Indians to whom the white man’s burden was devolved.^99^

What it meant to hold an instrument changed in other contexts, as the survey expanded and its “future Generations” came, in the absence of “half-caste” labor, to rely upon lower-caste “native agency.”^100^ We can see the shift by looking at the reception history of Thomas Hickey’s famous 1816 portrait of the surveyor and collector of South Indian antiquities, Colin Mackenzie, painted shortly after his elevation to Surveyor General of India, where he is flanked by three Indians (Figure 3a).^101^ Ancient and modern symbols are made to attach to two of the Indian figures. The darker-skinned attendant with the gray goatee proffers a palm-leaf manuscript. The assistant to the right (Mackenzie’s left) wears a satchel and holds a field surveying telescope, all the while gazing in evident adoration at Mackenzie. The assistant to Mackenzie’s right, the only Indian to meet the gaze of the viewer, carries nothing. Pictured in the background is the Buddhist stupa at Amaravati, which Mackenzie in 1798 mistook for a “Jain *deo*,” or godhead.^102^ Most historians who have considered this image have identified the three Indians (from the viewer’s left, respectively) as a Jain priest with whom Mackenzie had had contact, Dhurmia; Mackenzie’s Brahmin pandit and assistant, Kavali Venkata Lakshmayya; and Mackenzie’s “peon,” Kistnaji.^103^ Lakshmayya was one of two Telugu Brahmin brothers who assisted Mackenzie in compiling an archive and chronology for medieval South India, using their connections to acquire everything from lists of kings to coinage, down to the fourteenth century.

**Figure 3a.**
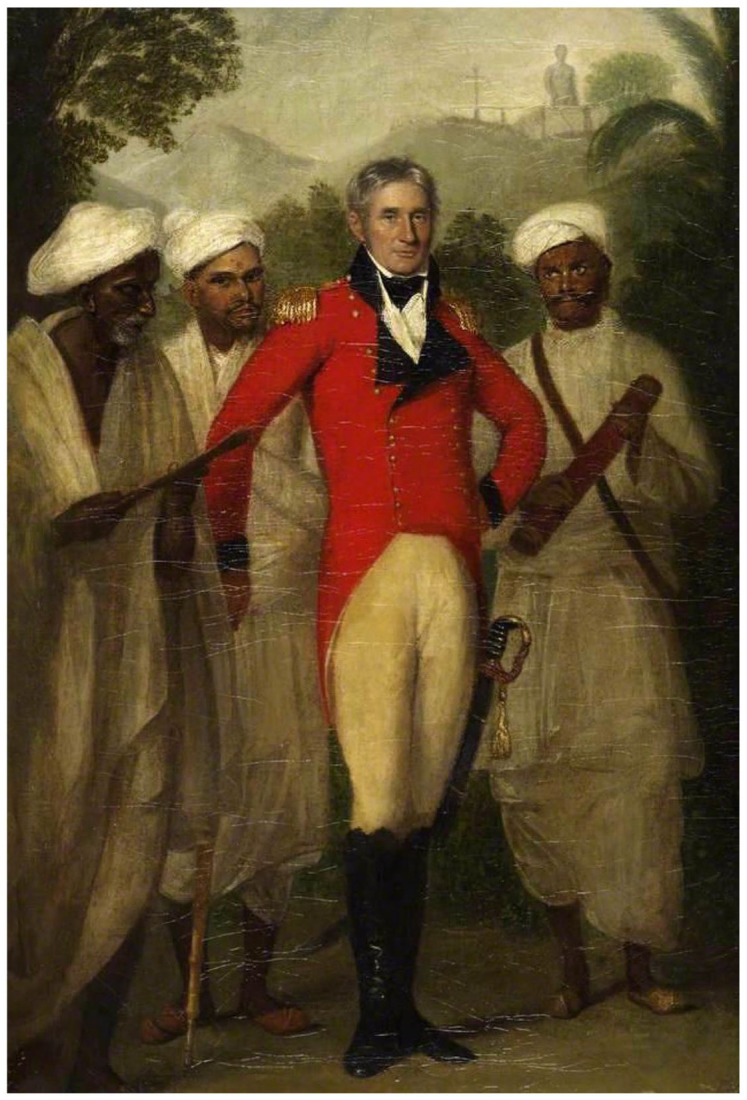
Thomas Hickey, Portait of Colin Mackenzie, Surveyor General of India. Calcutta, 1816. British Library, IOR F3.

**Figure 3b.**
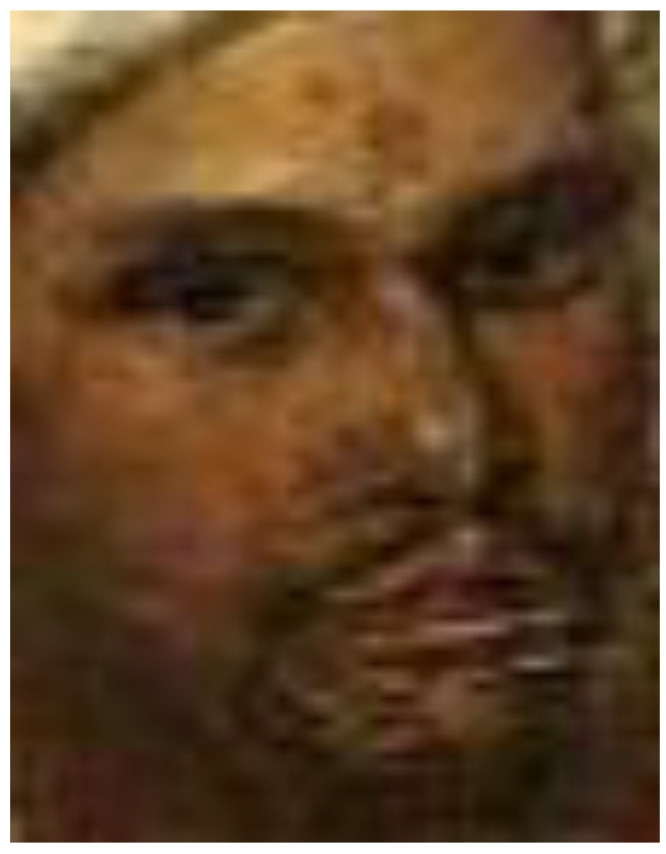
Detail of the man to Mackenzie’s right.

On the head of each Indian is visible what appears to be a caste-mark, a *tilak*. Devotees of Vishnu, or *Vaishnavites*, wear a *tilak* called an *urdhvapundra*, which consists of U-shaped vertical lines, sometimes with a central vertical line, while devotees of Shiva, called *Shaivaites*, wear the *tripundra*, made up of three white horizontal lines and a single central red mark.^104^ Contemporary Company officials were well aware of the significance of the *tilak*: an attempt to prohibit sepoys from wearing caste-marks is known to have contributed to the Vellore Mutiny in 1806.^105^

Historians have derived their identifications of Mackenzie’s assistants from Colonel Reginald Phillimore’s five-volume *Historical Records of the Survey of India*, the first appearing on the eve of independence, in 1945, and based on a number of (in some cases, no longer extant) archival collections. Phillimore cites the opinion of C. S. Srinivasachari and two other “leading Indian authorities” as to the identities of the Indians depicted in Hickey’s portrait. Srinivasachari surmises that the man to Mackenzie’s left is the “peon, Kistnaji, entrusted with the humble duty of carrying instruments.” He goes on to say that the figure to Mackenzie’s right is “an obvious *pandit*,” since he “has the caste mark of a Telugu Smartha Brahmin,” and on that basis “the most likely to be Kavali Venkata Lakshmaiah, of a Telinga family who in 1816 was still a young man.”^106^ Lakshmayya, together with his brother Boraya, used their connections with local Telugu elite to grant Mackenzie access to the village-level administrative records key to his archival project.^107^ The figure identified wears a single red mark with three smaller red dots above it. This is in fact not the caste-mark of any South Indian group.^108^ Moreover, Smartha Brahmins in contemporary Madras were nonsectarian Shaivaite laity, who did not usually wear caste-marks. When they did, it was the *tripundra*, not the *tilak* depicted in Figure 3b. So who are the figures depicted next to Mackenzie?

**Figure 4.**
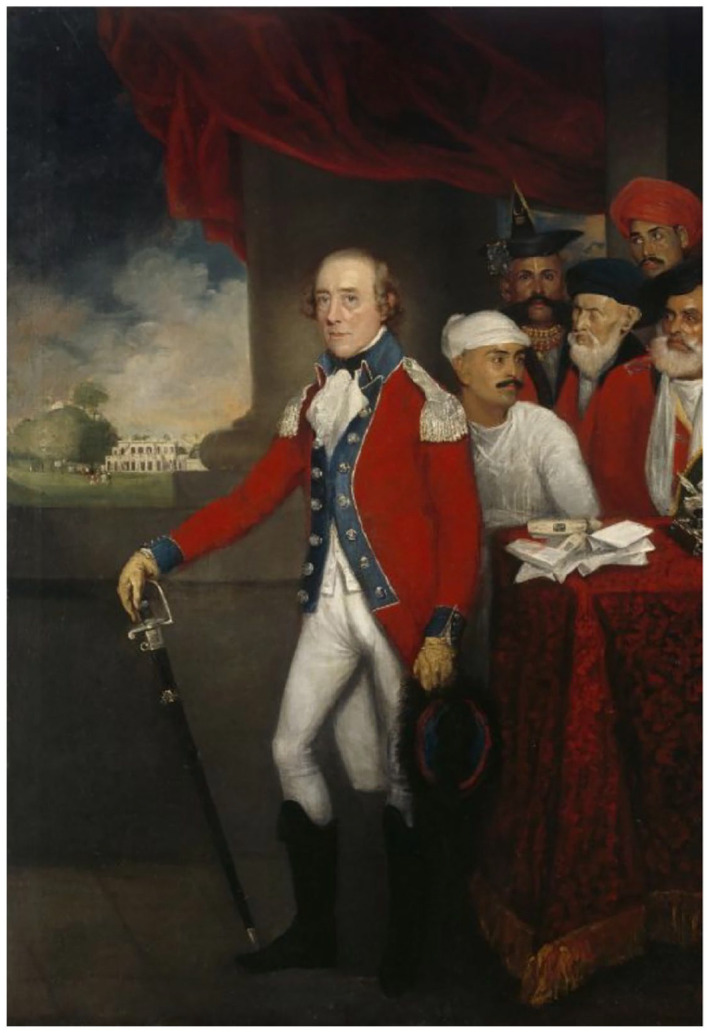
Thomas Hickey, Lieutenant-Colonel (later Major-General) William Kirkpatrick (1754–1812) with his assistants. National Gallery of Ireland, NGI.1860.

In all likelihood, the artist, Thomas Hickey, intended the Indian assistants to be mere generic types. Hickey, an Irishman who moved to South India with his two daughters permanently in 1798, aged fifty-eight, had earlier received a portrait commission from Lieutenant-Colonel William Kirkpatrick that depicted the latter with his Indian “assistants,” an improbable and incoherent collection of regional types (see Figure 4). Having failed to secure a position as the Company’s official portrait artist, he made a living from portraits such as Mackenzie’s.^109^ A note written on the back of the frame of Mackenzie’s portrait, presented in 1822 to the India House on Leadenhall Street, says it depicted Mackenzie together with “three distinguished Brahmins of the three leading sects in the south of India. The native holding the telescope is Kavelli Venkata Lakshmerjah.”^110^ In reality, Lakshmayya was not himself a surveyor. He is thought to be holding the telescope because of his elite status; in 1816, it can be reckoned a status symbol. By the time Phillimore is writing, in 1945, the sort of surveying labor the telescope signifies is of comparatively lower status, and allows Srinivasachari and Phillimore to see the figure as a “peon,” a gofer or fixer, usually of lower caste, merely carrying the instrument. The telescope, like the palm-leaf manuscripts, was an aspect of Mackenzie’s professional identity projected onto the “native agency” on which it was known to rely. The misrecognitions surrounding the identity of the Indian assistants occur because the significance of an instrument in the hands of an Indian flips. In 1816, the telescope signifies caste-bound prestige, but by 1945 it is so completely associated with subordinating labor that, despite being clothed similarly to the “obvious pandit,” even a “native observer” like Srinivasachari can no longer see a Brahmin bearing the instrument.^111^

## Conclusion

The observatory shows us that science under colonial rule was built around interventions into, as well as the appropriation of, certain forms of social order and historical memory. The inherent suitability of upper-caste scribal knowledge for observatory work had to do with the textual and computational nature of chronometric observation and maintenance. If by the end of the nineteenth century Indian observatory assistants were “machines without the certainty of machinery,” it was because they were viewed by astronomers as human replacements for self-registering instruments. In the observatory context, what began as scribal work eventually brought the Brahmins into analogy with mechanical recording and computing devices. But whereas in Greenwich, under Airy’s tenure, the division of labor meant observatory work came to bear resemblances to the factory and the accounts office, in colonial Madras, astronomy and accounting resembled each other at the level of practice because they were part of the same enterprise from the observatory’s inception.

Caste and race came into friction within the observatory because its social order was configured to support revenue surveying. Field surveys required intermediaries who could convincingly function as both Indian and European. Caste practices provided knowledge but also pedagogical modes, appropriated from the *tinnai*, that were used to teach both Indian and European knowledge. Under Topping’s original plan, the observatory’s social order was literally “half-caste.” Matters of race and trust soon became paramount, with astronomers according special importance and privilege to certain kinds of computation, to be carried out only by the descendants of Europeans. Yet the interpolations between race and caste this system required were unstable and short-lived. The closure of the Survey School meant the the work of the “half-caste” computers was reassigned to the Brahmins. No longer also teachers, the Brahmins’ everyday labor saw greater oversight and instrumentalization. Contemporary representations of Indians and instruments, like Mackenzie’s portrait and Goldingham’s pendulum experiments, show them as trustworthy and elite, yet subordinate, assistants to foregrounded Europeans. But very frequently, the Europeans were absent.

The real significance of Madras Observatory is therefore not only its incorporation of elite Indians into a new labor order and representational economy. Instrumental Brahmins are depicted when “half-castes” and the partial European agency they represent disappeared, and race subsumed caste in the everyday practice of scientific life in the observatory. Representations of caste were not always indexical, making issues of identity and attribution fraught, the result of the Company speaking, not always fluently, in an appropriated register. It is the dialogic construction of practice and representation that lends science under colonial rule its uncanny, polysemous character, simultaneously Indian and European, seemingly both ancient and modern.^112^

## Notes

1 See, for example, Kerby C. Alvarez, “Instrumentation and Institutionalization: Colonial Science and the Observatorio Meteorológico de Manila, 1865–1899,” *Philippine Studies Historical & Ethnographic Viewpoints* 64.3–4 (2016): 385–416; Simon Schaffer, “Keeping the Books at Parramatta Observatory,” in David Aubin, Charlotte Bigg and H. Otto Sibum (eds.), *The Heavens on Earth: Observatories and Astronomy in Nineteenth Century Science and Culture* (Durham, NC: Duke University Press, 2010).

2 Papers of George Biddell Airy, Royal Greenwich Observatory Archives, Cambridge University Library, RGO 6/154, File 13, p.358; On Doberck’s labor regime at Hong Kong Observatory, and observatories as places of work in the colonial context more broadly, see Fiona Williamson, “Just Doing Their Job: The Hidden Meteorologists of Colonial Hong Kong c. 1883–1914,” *The British Journal for the History of Science* 54.3 (2021): 341–59; Cole Roskam, “Constructing Climate: The Hong Kong Observatory and Imperial Britain’s Meteorological Networks, 1842–1912,” *The Journal of Architecture* 26.8 (2021): 1241–70.

3 N. R. Pogson to C. R. Markham, February 22, 1871. British Library (hereafter BL) MSS Eur. B251.

4 N. R. Pogson to Airy, October 1886. *Papers of George Airy*, RGO 6/154, p.194.

5 On class, discipline, and labor under George Airy’s Greenwich, see Simon Schaffer, “Astronomers Mark Time: Discipline and the Personal Equation,” *Science in Context* 2.1 (1988): 115–45; Mary Croarken, “Astronomical Labourers: Maskelyne’s Assistants at the Royal Observatory, Greenwich, 1765-1811,” *Notes and Records of the Royal Society of London* 57 (2003): 285–98; Scott Alan Johnston, “Managing the Observatory: Discipline, Order and Disorder at Greenwich, 1835–1933,” *The British Journal for the History of Science* 54.2 (2021): 155–75; Daniel Belteki, “The Spring of Order: Robert Main’s Management of Astronomical Labor at the Royal Observatory, Greenwich,” *History of Science* (2021): 1–19. (Journal pre-proof available online 20 July 2021).

6 See Projit Bihari Mukharji, “Darwin’s Bulbuls: South Asian Cultures of Bird Fighting and Darwin’s Theory of Sexual Selection,” *BJHS Themes* 6 (2021): 63–79.

7 On the history of science and globalism, see Helen Tilley, “Global Histories, Vernacular Science, and African Genealogies; or, Is the History of Science Ready for the World?” *Isis* 101.1 (2010): 110–11; Sujit Sivasundaram, “Sciences and the Global: On Methods, Questions, and Theory,” *Isis* 101.1 (2010): 146–58; Carla Nappi, “The Global and Beyond: Adventures in the Local Historiographies of Science,” *Isis* 104.1 (2013): 102–10; on braiding as a metaphor for the interaction of knowledge traditions under colonial rule, see Projit Bihari Mukharji, *Doctoring Traditions: Ayurveda, Small Technologies, and Braided Sciences* (Chicago: University of Chicago Press, 2016), pp.26–7.

8 Bruno Latour, *Reassembling the Social: An Introduction to Actor-Network Theory* (Oxford: Oxford University Press, 2005), p.44; Kapil Raj, “Beyond Postcolonialism . . . and Postpositivism: Circulation and the Global History of Science,” *Isis* 104.2 (2013): 337–47; Simon Schaffer et al. (eds.), *The Brokered World: Go-Betweens and Global Intelligence, 1770-1820* (Sagamore Beach, MA: Science History Publications, 2009).

9 On what Kapil Raj calls “open-air” scientific practices in the colony, see *Relocating Modern Science* (New York: Palgrave, 2006), pp.14; 231–2. See also Simon Schaffer, “Instruments and Ingenuity between India and Britain,” *Bulletin of the Scientific Instrument Society* 140 (2019): 2–13; Thomas Simpson, *The Frontier in British India: Space, Science, and Power in the Nineteenth Century* (Cambridge: Cambridge University Press, 2021).

10 Paolo Palladino and Michael Worboys, “Science and Imperialism,” *Isis* 84.1 (1993): 91–102; On the links between colonial rule and astronomy and physics, see Simon Schaffer, “Exact Sciences and Colonialism: Southern India in 1900,” in Moritz Epple and Claus Zittel (eds.), *Science as Cultural Practice*, Vol. 1 (Berlin: Akademie Verlag, 2010), pp.121–40.

11 See in particular Christopher John Fuller and Haripriya Narasimhan, *Tamil Brahmins: The Making of a Middle-Class Caste* (Chicago: University of Chicago Press, 2014); Ajantha Subramanian, *The Caste of Merit: Engineering Education in India* (Cambridge, MA: Harvard University Press, 2019).

12 Stephen Evans, “Macaulay’s Minute Revisited: Colonial Language Policy in Nineteenth-Century India,” *Journal of Multilingual and Multicultural Development* 23.4 (2002): 260–81; Catriona Ellis, “Education for All: Reassessing the Historiography of Education in Colonial India,” *History Compass* 7.2 (2009): 363–75; Parimala V. Rao, *Beyond Macaulay: Education in India, 1780–1860* (New York: Routledge, 2020), pp.71–94.

13 The observatory should be seen as part of the Company “writing machine” in eighteenth century South India. See Bhavani Raman, *Document Raj: Writing and Scribes in Early Colonial South India* (Chicago: University of Chicago Press, 2012).

14 Ravi Ahuja, “Labour Relations in an Early Colonial Context: Madras, c. 1750–1800,” *Modern Asian Studies* 36.4 (2002): 793–826.

15 The term “half-caste” is usually considered a pejorative, and during the period under study, referred most often to children born to Indian mothers, of European fathers.

16 Madras was, however, not the first observatory constructed by Europeans in India. In 1787, the Danish Crown funded the construction of a chronometric observatory in the tower of the Church of Zion, in the missionary settlement of Tranquebar. See Niklas Thode Jensen, “The Tranquebarian Society: Science, Enlightenment and Useful Knowledge in the Danish-Norwegian East Indies, 1768–1813,” *Scandinavian Journal of History* 40.4 (2015): 535–61.

17 Michael Topping, “Correspondence respecting Topping’s Nautical Surveys and the Foundation of Madras Observatory,” BL IOR/X/3948, 1790, pp.1–7.

18 See William Petrie, “Memorandum respecting the Observatory Delivered to the Honourable Sir George Barlow, Governor of Ft. St. George,” September 4th, 1808, Madras Manuscript Records (1794–1812), Indian Institute of Astrophysics, Bangalore (hereafter Madras MS Records, IIA), pp.73–86. In 1808, Barlow had begun a program of cost-cutting, and the observatory was forced to justify itself. Petrie’s intercession was crucial as he sat on the governor’s council and had first begun astronomical observations at Egmore in Madras in 1787. The Madras MS Records contain selected correspondence between company astronomers Michael Topping, John Goldingham, and John Warren, and cover a period from January 5th 1794 to July 1st 1812. They are available digitally via the IIA Institutional Repository, <http://prints.iiap.res.in/handle/2248/998> (accessed December 21, 2021). See also the Madras Manuscript Observations (1795–1800), Indian Institute of Astrophysics, Bangalore, <http://prints.iiap.res.in/handle/2248/991>.

19 On Guldenheim/Goldenham’s Danish roots, see S. M. Razaullah Ansari, *Introduction of Modern Western Astronomy in India During 18-19 Centuries* (New Delhi: Department of History of Medicine and Science, Institute of History of Medicine and Medical Research, 1985), p.25; Edwin Danson, *Weighing the World: The Quest to Measure the Earth* (Oxford: Oxford University Press, 2009), p.206.

20 See 1800–1815, BL IOR/E/4/880, 1792, pp.386–88. Fort St. George opined that “the appointment of a Bramin assistant may be useful in propagating knowledge amongst the Natives and in obtaining information of such parts as it may not be expedient for European Surveyors to visit.

21 R. H. Phillimore, *Historical Records of the Survey of India*, Vol. 2 (Dehra Dun: The Offices of the Survey of India, 1950), p.196.

22 They also were to “keep the Instruments in proper condition and Order, . . . being responsible to [the Company astronomer] and the public for them.” John Warren to Colin Mackenzie, Surveyor General of Madras, July 4th, 1811, Madras MS Records, IIA.

23 David Aubin, “On the Epistemic and Social Foundations of Mathematics as Tool and Instrument in Observatories, 1793–1846,” in Johannes Lenhard and Martin Carrier (eds.), *Mathematics as a Tool: Tracing New Roles of Mathematics in the Sciences* (Cham: Springer, 2017), pp.177–96.

24 See Bernard Cohn, “The Command of Language and the Language of Command,” *Subaltern Studies* 4 (1985): 276–329; Michael S. Dodson, “Translating Science, Translating Empire: The Power of Language in Colonial North India,” *Comparative Studies in Society and History* 47.4 (2005): 809–35.

25 On the recuitment of pandits, see Rosane Rocher, “The Career of Rādhākānta Tarkavāgīśa, an Eighteenth-Century Pandit in British Employ,” *Journal of the American Oriental Society* 109.4 (1989): 627–33; Brian A. Hatcher, “Indigent Brahmans, Industrious Pandits: Bourgeois Ideology and Sanskrit Pandits in Colonial Calcutta,” *Comparative Studies of South Asia, Africa and the Middle East* 16.1 (1996): 15–26. On William Jones’ influences, see Alun David, “Sir William Jones, Biblical Orientalism and Indian Scholarship,” *Modern Asian Studies* 30.1 (1996): 173–84.

26 Thomas Trautmann, “Hullabaloo about Telugu,” *South Asia Research* 19.1 (1999): 53–70; Thomas Trautmann, *Languages and Nations: The Dravidian Proof in Colonial Madras* (Berkeley: University of California Press, 2006).

27 Thomas Trautmann (ed.), *The Madras School of Orientalism: Producing Knowledge in Colonial South India* (New Delhi: Oxford University Press, 2009), p.2.

28 Marine chronometers that did not require rewinding were simply too unreliable and expensive to be adopted on most ships until the 1840s. See Morgan Kelly, Cormac Ó Gráda, and Peter M. Solar, “Safety at Sea during the Industrial Revolution,” *The Journal of Economic History* 81.1 (2021): 239–75.

29 By 1821, under Goldingham, “the comparisons of chronometers alone [occupied] 10 folio volumes.” See “The Company’s Astronomer at Madras, John Goldingham, is Directed to Furnish the Surveyor General of India, with Any Information the Latter Requires,” BL IOR/F/4/707/19162, p.33.

30 On maritime chronometers and the East India Company, see Andrew S. Cook, “Alexander Dalrymple and John Arnold: Chronometers and the Representation of Longitude on East India Company Charts,” *Vistas in Astronomy* 28 (1985): 189–95; David Philip Miller, “Longitude Networks on Land and Sea: The East India Company and Longitude Measurement ‘in the Wild’, 1770–1840,” in Richard Dunn and Rebekah Higgitt (eds.), *Navigational Enterprises in Europe and its Empires, 1730–1850* (New York: Springer, 2015), pp.223–47. On maintenance as a key analytic category in the history of technology, see Andrew L. Russell and Lee Vinsel, “After Innovation, Turn to Maintenance,” *Technology and Culture* 59.1 (2018): 1–25; Aditya Ramesh, “The Value of Tanks: Maintenance, Ecology and the Colonial Economy in Nineteenth-Century South India,” *Water History* 10.4 (2018): 267–89.

31 On the *kanakkupillai*, see Bhavani Raman, “Document Raj: Scribes and Writing Under Early Colonial Rule Madras, 1771-1860” (PhD diss., University of Michigan, 2007), pp.31ff.

32 See Bhavani Raman, “The Familial World of the Company’s *kacceri* in Early Colonial Madras,” *Journal of Colonialism and Colonial History* 9.2 (2008). https://muse.jhu.edu/article/246576

33 These were diasporic groups who migrated from what is now Telangana and Maharashtra between the fifteenth and the seventeenth centuries and comprised part of the service gentry in the administrations of the *nayaks* [rulers] of Madurai and Thanjavur. See Robert Eric Frykenberg, “Elite Groups in a South Indian District: 1788-1858,” *The Journal of Asian Studies* 24.2 (1965): 261–81.

34 On the complexities of caste, kinship, and language within the *kacceri*, see Raman, “Document Raj,” pp.57–70 (note 31).

35 Susan Neild-Basu, “The Dubashes of Madras,” *Modern Asian Studies* 18.1 (1984): 1–31.

36 On *dubashes* and company officials and issues of practice in the *kacceri*, and for a general account of the company’s Madras revenue bureaucracy, see Raman, *Document Raj*, pp.14; 23–52 (note 13).

37 U. Kalpagam, “Cartography in Colonial India,” *Economic and Political Weekly* 30.30 (1995): 87–98; Bernardo A. Michael, “Making Territory Visible: The Revenue Surveys of Colonial South Asia,” *Imago Mundi* 59.1 (2007): 78–95.

38 More precise mapping allowed the company to detect what it called “hidden cultivation.” See Nilmani Mukherjee, *The Ryotwari System in Madras, 1792-1827* (Calcutta: Firma K.L. Mukhopadhyay, 1962), pp.130ff.

39 In practice this order was rarely followed, and by 1814 the observatory was placed under the authority of the expanding survey. See BL IOR/E/4/880, p.392, and Matthew H. Edney, *Mapping an Empire: The Geographical Construction of British India, 1765-1843* (Chicago: University of Chicago Press, 1999), pp.172–5 on the relationship between the observatory and the revenue survey.

40 Munro’s system was short-lived: by 1822, under-reporting and inefficiency, as well as problems with scaling operations Presidency-wide, meant that Company collectors relied on the records of *kanakkupillais*. See Chapter 6, on the survey, in Mukherjee, *The Ryotwari System*, pp.125–49 (note 37). See also David Ludden, *Peasant History in South India* (Princeton, NJ: Princeton University Press, 1985), pp.107–15.

41 E. W. Maunder, a former assistant to Airy at Greenwich, wrote in 1900 that the astronomer’s “life, on the one side approximates to that of the engineer; on the other, to that of the accountant.” *The Royal Observatory Greenwich: A Glance at its History and Work* (London: 1900), p.15, as cited in William J. Ashworth, “The Calculating Eye: Baily, Herschel, Babbage and the Business of Astronomy,” *The British Journal for the History of Science* 27.4 (1994): 409–41.

42 According to Maunder, a visitor to Greenwich would encounter “official rooms not unlike those of Somerset House or Whitehall, and its occupants for the most part similarly engaged in what is, apparently, merely clerical work. An examination of the big folios would of course show that instead of being ledgers of sales of stamps, or income tax schedules, they referred to stars, planets, and sunspots.” See Maunder, *Greenwich*, p.137, as cited in Robert W. Smith, “A National Observatory Transformed: Greenwich in the Nineteenth Century,” *Journal for the History of Astronomy* 45 (1991): 5–20.

43 Dhruv Raina, “The European Construction of ‘Hindu Astronomy’ (1700–1900),” in Knut A. Jacobsen and Ferdinando Sardella (eds.), *Handbook of Hinduism in Europe* (2 Vols.) (Leiden: Brill, 2020), pp.123–51; Simon Schaffer, “The Asiatic Enlightenments of British Astronomy,” in S. Schaffer, Lissa L. Roberts, K. Raj, and J. Delbourgo (eds.), *The Brokered World: Go-Betweens and Global Intelligence, 1770–1820* (Sagamore Beach, MA: Science History Publications, 2009), pp.49–104.

44 See Andrew Scott, “Memorandum,” January 1st, 1810, Madras MS Records, IIA, pp.99–101. The observatory’s interest in *jyotiśāstra* seems to have been mostly antiquarian in nature. But the Brahmins’ capacity for astronomical computation largely transferred from the mathematical skillset required for *jyotiśāstra.*

45 Dharma Kumar, “The Forgotten Sector: Services in Madras Presidency in the First Half of the Nineteenth Century,” *The Indian Economic & Social History Review* 24.4 (1987): 367–93.

46 Colaba in the 1840s employed “two Assistant Brahmins” and a Parsi as an assistant computer. See Joydeep Sen, *Astronomy in India, 1784–1876* (London: Routledge, 2015), p.100.

47 As Babu has noted, “situations that the *vettiyān* (village assistant who measured land) and the *nīrkāran* (water regulator) lived with and cognitively engaged with as part of their manual work were subjects of learning within the institution, in which they and their children could never set foot.” See Senthil Babu, “Memory and Mathematics in the Tamil *tinnai* Schools of South India in the Eighteenth and Nineteenth Centuries,” *The International Journal for the History of Mathematics Education* 2.1 (2007): 15–37.

48 See Robert E. Frykenberg, “Modern Education in South India, 1784-1854: Its Roots and Its Role as a Vehicle of Integration under Company Raj,” *The American Historical Review* 91.1 (1986): 37–65; Robert E. Frykenberg, “The Myth of English as a ‘Colonialist’ Imposition upon India: A Reappraisal with Special Reference to South India,” *Journal of the Royal Asiatic Society* 120.2 (1988): 305–15.

49 Phillimore, *Historical Records*, Vol. 2, p.348 (note 21). Orphans would become a major source of colonial labor in nineteenth century British India. See David Arnold, “European Orphans and Vagrants in India in the Nineteenth Century,” *The Journal of Imperial and Commonwealth History* 7.2 (1979): 104–27; Satadru Sen, “The Orphaned Colony: Orphanage, Child and Authority in British India,” *The Indian Economic & Social History Review* 44.4 (2007): 463–88.

50 Jana Tschurenev, “Diffusing Useful Knowledge: The Monitorial System of Education in Madras, London and Bengal, 1789–1840,” *Paedagogica Historica* 44.3 (2008): 245–64; Simon Schaffer, “How Disciplines Look,” in Andrew Barry and Georgina Born (eds.), *Interdisciplinarity* (London: Routledge, 2013), pp.73–97.

51 Carl H. Nightingale, “Before Race Mattered: Geographies of the Color Line in Early Colonial Madras and New York,” *The American Historical Review* 113.1 (2008): 48–71.

52 Michael Topping to David Haliburton, President of the Board of Revenue, Ft. St George. January 5th, 1794, Madras MS Records, IIA, p.4.

53 “[T]he rank of these children, the hold you have over them by the mode of education and discipline . . . all is calculated to render them valuable to this settlement and subservient to the general good. They are instruments in your hands, fitted for your hands, and no other, and can no ways fail you.” Andrew Bell, *An Experiment in Education, Made at the Male Asylum of Madras* (London: Cadell & Davies, 1797), pp.4–7; 33.

54 Topping to Haliburton, Madras MS Records, IIA, p.5 (note 52).

55 Given Warren’s frequent absences, “always” is probably an overstatement. John Warren to Ft. St. George, June 4th, 1811, Madras MS Records, IIA, partially reproduced in Phillimore, *Historical Records*, Vol. 2, p.196 (note 21).

56 Matthew H. Edney, *Mapping an Empire*, pp.186ff.

57 It’s possible that Scott’s scheme was merely an attempt to entrap Srinivasacharya, and have him fired. However, it’s also probable that the instruments could have been fenced; in Madras, there was a fairly extensive collectors’ market for natural-philosophical equipment. In the first few decades of the nineteenth century, the Madras Courier regularly listed advertisements for air-pumps, electrifying machines, and other “Medical and Philosophical apparatus.” Savithri Preetha Nair, *Raja Serfoji II: Science, Medicine and Enlightenment in Tanjore* (London: Routledge India, 2014), p.90.

58 Srinivasacharya Braminy to John Warren, May 4th, 1811, Madras MS Records, IIA, pp.145–9.

59 The pagoda was a gold coin used in southern India, usually valued at seven shillings, or £0.35. For information on observatory and surveyors’ salaries, see Madras MS Records, IAA, p.32–4; 74.

60 An analogy might be made with the women hired as computers at ballistics laboratories during World War II: “Ballistics computing, a man’s job during WW1 was feminized by WW2.” Jennifer S. Light, “When Computers Were Women,” *Technology and Culture* 40.3 (1999): 455–83, 460.

61 Thomas Glanville Taylor, *Result of Astronomical Observations Made at the Honorable East India Company’s Observatory at Madras*, Vol. 1 (1831), (Madras: Orphan Asylum Press, 1832), p.i. The quote therein is from George Biddell Airy, *Astronomical Observations Made at the Observatory of Cambridge*, Vol. 1 (1828), (Cambridge: J. Smith, 1829), pp.v–vi.

62 Taylor, *Astronomical Observations*, Vol. 1, p.30 (note 61).

63 Thomas Glanville Taylor, *Result of Astronomical Observations Made at the Honorable East India Company’s Observatory at Madras*, Vol. 2 (1832), (Madras: Orphan Asylum Press, 1833), p.iii.

64 Babu, “Memory and Mathematics,” 24 (note 47). Whether the Brahmins used specific techniques that were different from the Tamil arithmetic taught in the *tinnai* in reducing observations is uncertain, given their mnemonic nature. Nevertheless, mathematical education in the *tinnai* emphasized carrying out fundamental operations like addition, subtraction, multiplication, and division with the kind of speed and accuracy also required in the observatory.

65 See the discussion in Mukharji, *Doctoring Traditions*, p.92 (note 7).

66 George Airy, “Address Delivered by the President, G.B. Airy, Esq. F.R.S., Astronomer Royal, on Presenting the Gold Medal of the Society to Mr. Charles Rümker,” *Monthly Notices of the Royal Astronomical Society* 14 (1854): p.145.

67 See “Sienvascharry Bramin, Head Assistant in Observatory, Pensioned and Granted Palanquin and Allowance,” BL fIOR/E/4/943, 1830, pp.438–9.

68 Taylor, *Astronomical Observations* Vol. 4 (1836 & 1837), (Madras: R.W. Thorpe, Vepery Mission Presss, 1838), p.27.

69 Steven Shapin, “The Invisible Technician,” *American Scientist* 77.6 (1989): 554–63.

70 Taylor, *Astronomical Observations* Vol. 3 (1834 & 1835), (Madras: Male Asylum and Vepery Mission Presses, 1836), p.3.

71 Taylor, *Astronomical Observations* Vol. 1, pp.4ff (note 60).

72 In Madras, unlike in Bengal, there was a “preference for middle and low-caste soldiers . . . military service was not the preserve of high-caste communities in south India.” Vikram Harijan, “Occupational Caste Groups and Madras Army in Colonial India,” *Proceedings of the Indian History Congress* 72 (2011): 642–9.

73 The modern practice of painting *tilaks* on instruments has to do with *Ayhudhapuja*, a festival in which machines are worshipped, described by some scholars as part of the Brahminization of scientific work in modern India. See Renny Thomas, “Brahmins as Scientists and Science as Brahmins’ Calling: Caste in an Indian Scientific Research Institute,” *Public Understanding of Science* 29.3 (2020): 306–18.

74 The *tilak* in Figure 1 is the Shaivite equivalent of that visible on Srinivasacharya’s head in Goldingham’s 1822 image. See for example BL Add Or 4385, which depicts a “Savia Brahmin,” c.1850, “marked with three parallel lines of white ash on forehead and upper body, and carrying a manuscript. He also has a red bindu under the forehead mark.”

75 Guilliame Le Gentil, *Voyage dans les mers de l’Inde*, 2 Vols. (Switzerland: Libraires Associés, 1780), quoted in Helen Sawyer Hogg, “Out of Old Books (Le Gentil and the Transits of Venus, 1761 and 1769 Continued, with Plate V),” *Journal of the Royal Astronomical Society of Canada* 45 (1951): 127; Michel Danino, “Le Gentil à Pondichéry: de Vénus à l’Inde,” *Synergies Inde* 7 (2016): 39–41; See also the discussion of Le Gentil at the end of Otto Neugebauer, “Tamil Astronomy: A Study in the History of Astronomy in India,” *Osiris* 10 (1952): 252–76.

76 See the references to an astrolabe “in the *Nagry* character” mentioned in Reuben Burrow, “A Proof that the Hindoos had the Binomial Theorem,” *Asiatic Researches* 2 (1790): 487.

77 William Hunter, “Some Account of the Astronomical Labours of Jayasinha, Rajah of Ambere, or Jayanagar,” *Asiatic Researches* 5 (1797): 177–211.

78 The story was well-known enough to be casually referenced as late as 1854, the Brahmin who smashed the telescope seen in analogy with those who would not look into Galileo’s telescope. See “On the Utility of the Mathematics,” *Mechanics Magazine* 61 (1854): 173.

79 For the anecdote see James Forbes, *Oriental Memoirs*, Vol. 2 (London: T. Bensley, 1813), pp.468ff.

80 John Goldingham, “Observations for Ascertaining the Length of the Pendulum at Madras in the East Indies,” *Philosophical Transactions of the Royal Society of London* 112 (1822): 127–70.

81 Phillimore, *Historical Records*, Vol. 2, p.410 (note 21).

82 A. D. C. Simpson, “The Pendulum as the British Length Standard: A Nineteenth-Century Legal Aberration,” in *Making Instruments Count: Essays on Historical Scientific Instruments* (London: Ashgate, 1993), 174–90.

83 See Raj, *Relocating Modern Science*, pp.181–222 (note 9).

84 Henry Kater, “An Account of Experiments for Determining the Length of the Pendulum Vibrating Seconds in the Latitude of London,” *Philosophical Transactions of the Royal Society of London* 108 (1818): 33–102.

85 Charles Babbage would later heap scorn upon gravimetric pendulum experimenters’ methods, noting that summed errors often brought “discordant observations into an almost unprecedented accordance” with anticipated results. See his *Reflections on the Decline of Science in England: And on Some of Its Causes* (London: B. Fellowes, 1830), pp.75–84.

86 Goldingham, “Observations,” p.127 (note 80).

87 Kater, “An Account” (note 84).

88 See the discussion in Sujit Sivasundaram, *Waves across the South: A New History of Revolution and Empire* (London: William Collins, 2021), pp.245–60.

89 See the images of the Latin plaque in R. C. Kapoor, “Comet Tales from India: The Great September Comet of 1882 (C/1882 R1),” *Journal of Astronomical History and Heritage* 23.2 (2020): 353–74.

90 Michael Topping, *Description of an Astronomical Observatory Erected at Madras in 1792*, Royal Astronomical Society (hereafter RAS) MSS Madras 2, December 1792, pp.5v–6r.

91 See Eugene F. Irschick, *Dialogue and History: Constructing South India, 1795-1895* (Berkeley: University of California Press, 1994), pp.97–100, 8–15; Mikhail M. Bakhtin, *The Dialogic Imagination: Four Essays*, ed. Michael Holquist, trans. Caryl Emerson and Michael Holquist (Austin: University of Texas Press, 1981), pp.324, 351. On land grant stelae, see Kathleen D. Morrison and Mark T. Lycett, “Inscriptions as Artifacts: Precolonial South India and the Analysis of Texts,” *Journal of Archaeological Method and Theory* 4.3–4 (1997): 215–37. See for example those described in Heinrich Lüders, “Two Pillar Inscriptions of the Time of Krishnaraya of Vijayanagara,” *Epigraphia Indica* 6 (1900): 108–33.

92 Topping continues, making the temple connection explicit: “This kind of stone takes a fine black polish, as I myself have witnessed in many of the Indian Temples; but to bring it to so desirable a degree of beauty requires great labor, and very considerable expense.” Topping, *Description*, pp.3r–3v (note 90).

93 Ibid., p.1v.

94 Bernard S. Cohn, *Colonialism and Its Forms of Knowledge: The British in India* (Princeton, NJ: Princeton University Press, 1996), p.53.

95 None of the observatory structures remain except for the pillar. Were it not for an 1802 inscription marking the pillar a geodetic baseline for surveyors, the site, now a museum, could at first glance pass for something ancient. The observatory was closed in 1899, but see the photos of the plaque and the transit pillar in R. K. Kochhar, “Madras Observatory-Buildings and Instruments,” *Bulletin of the Astronomical Society of India* 13 (1985): 287–302.

96 G. V. Juggarow, “Observations Respecting Halley’s Comet,” in *The Madras Journal of Literature and Science*, Vol. 1 (Madras: Vepery Mission Press, 1834), pp.62–72.

97 G. V. Juggarow, “On the Mass of the Planet Jupiter,” in J. C. Morris (ed.), *The Madras Journal of Literature and Science*, Vol. 3 (Madras: Vepery Mission Press, 1836), pp.131–3.

98 The most notable being the Raja of Travancore, Swathi Thirunal. See Jessica Ratcliff, “Travancore’s Magnetic Crusade: Geomagnetism and the Geography of Scientific Production in a Princely State,” *The British Journal for the History of Science* 49.3 (2016): 325–52, and the references in Sen, *Astronomy in India*, pp.75ff (note 46).

99 Robert Cole (ed.), *The Madras Journal of Literature and Science*, Vol. 4 (Madras: Vepery Mission Press, 1836), p.133. In 1840, Juggarow set up a private observatory in Vizagapatam, with his family’s financial backing. The observatory was equipped with a transit circle by Troughton and an astronomical regulator, which appear to have been used in the determination of the longitude of Vizagapatam. Few other records of the observatory and its activities remain. Juggarow himself died in 1856, at the early age of thirty-nine, whereupon his observatory was taken over by his son-in-law. See N. Kameswara Rao, A. Vagiswari, and Christina Birdie, “Early Pioneers of Telescopic Astronomy in India: GV Juggarow and His Observatory,” *Current Science* 100.1 (2011): 1575–81.

100 The work of Indian surveyors, referred to as “Native agency,” was both labor-saving and suspect. This meant that the “strictest surveillance is necessary on the part of Assistants to guard against the great negligence in this respect [the maintenance of theodolites] generally prevalent amongst Native Surveyors.” See Ralph Smyth and Henry Thuillier, *A Manual of Surveying for India* (Calcutta: W. Thacker & Co., 1851), p.135.

101 Phillimore, *Historical Records of the Survey of India*, 1815 to 1830, Vol. 3 (Dehra Dun: The Offices of the Survey of India, 1954),

102 Mackenzie was the first European to realize Jains were a distinct religious group. Jennifer Howes, “Colin Mackenzie and the Stupa at Amaravati,” *South Asian Studies* 18.1 (2002): 53–65.

103 See for example Raj, *Relocating Modern Science*, p.219 (note 9) and Edney, *Mapping an Empire*, p.154 (note 39).

104 The most complete record of contemporary caste marks in South India are thirty-two watercolors by an artist of the Moochy caste, commissioned by the Fort Adjutant at Vellore c.1828. See BL Add Or 39–70, and in particular 54, which depicts “Shaiva and Vaishnava Brahmins with their wives, which is inscribed: ‘‘Sheeva’ Bramin’;’ ‘Vishnoo’ Bramin’;’ Bramin ‘Sheeva’. Such as missionaries might call impracticables.’” <www.bl.uk/manuscripts/FullDisplay.aspx?ref=Add_Or_54> (accessed March 4, 2021).

105 See K. K. Pillay, “The Causes of the Vellore Mutiny,” *Proceedings of the Indian History Congress* 20 (1957): 306–11.

106 Phillimore, *Historical Records*, Vol. 2, plate 22, facing p.427 (note 21).

107 On Mackenzie’s recruitment of Telugu Niyogi Brahmins, and the distinction between Sanskrit and local knowledge in South India, see Velcheru Narayana Rao, “Print and Prose: Pandits, Karanams and the East India Company in the Making of Modern Telugu,” in Stuart Blackburn and Vasudha Dalmia (eds.), *India’s Literary History: Essays on the Nineteenth Century* (New Delhi: Permanent Black, 2004).

108 For a description of the Telugu Smartha Brahmin caste mark, see C. Ramachendrier, *Collection of the Decisions of the High Courts and the Privy Council on the Hindu Law of Marriage* (Madras: V. Kalyanaram Iyer, 1891), p.35. Neither contemporaneous portraiture nor detailed breakdowns of South Indian caste marks and proscriptions collated by Ramachendrier mention anything like the caste mark on the forehead of the man to Mackenzie’s right.

109 Rama Mantena, *The Origins of Modern Historiography in India: Antiquarianism and Philology, 1780-1880* (New York: Palgrave, 2012), pp.78ff.

110 William Foster, *A Descriptive Catalogue of the Paintings, Statues, &c., in the India Office* (London: HM Stationery Office, 1924), p.7.

111 Interpretations of the image hinge on whether the Brahmin or the peon holds the telescope. For Lakshmayya wielding a status symbol, see Jennifer Howes, “Colin Mackenzie with His Three Indian Assistants,” *The British Art Journal* 20.2 (2019): 53–7. For Kistnaji holding the telescope as a metonym for colonial domination, see Subhash Jaireth, “Close Encounters of the Colonial Kind or Looking for Colonel Mackenzie’s Pandits,” *Social Alternatives* [Special Issue: Painting Politics edited by Roland Bleiker] 20.4 (2001): 55–60.

112 The same has been said of science in modern India, in part because the independent nation “meant not a negation of the colonial configuration of the territory and its people but their reinscription under the authority of science.” If “India appears simultaneously as something altogether new and unmistakably old,” it is because colonial polysemy is carried forward by the postcolonial state. See Gyan Prakash, *Another Reason: Science and the Imagination of Modern India* (Princeton, NJ: Princeton University Press, 1999), pp.7–14.

